# Prevalence of voice & swallowing complaints in Pre-operative thyroidectomy patients: a prospective cohort study

**DOI:** 10.1186/s40463-014-0028-4

**Published:** 2014-07-15

**Authors:** Theresa Holler, Jennifer Anderson

**Affiliations:** 1 Department of Otolaryngology – Head & Neck Surgery, St. Michael’s Hospital, University of Toronto, 30 Bond Street, 8C-129, Toronto, M5B 1 W8 ON Canada; 2 Voice Disorders Clinic, St. Michael’s Hospital, University of Toronto, 30 Bond Street, 8 Cardinal Carter North, Toronto, M5B 1 W8 ON Canada

**Keywords:** Surgery, Outcomes, Voice, Swallowing, Thyroid

## Abstract

**Background:**

Compressive symptoms are common in patients with thyroid disease and are an accepted indication for thyroidectomy. The objective of this study was to evaluate the prevalence and severity of voice and swallowing complaints in pre-operative thyroidectomy patients and to compare these with thyroid volume, thyroid pathology and laryngopharyngeal reflux.

**Methods:**

A consecutive case series design was performed. All patients undergoing a thyroidectomy (partial or total) at a tertiary care centre during a 2 year period were invited to participate. Fifty nine (10 male, 49 female) aged 19–73 were enrolled (59.3% total thyroidectomy, 40.7% partial thyroidectomy). Voice and swallowing complaints were subjectively evaluated using the Voice Handicap Index (VHI) and the Modified Swallowing Impairment Score (MSIS), respectively. All patients completed the Reflux Symptom Index (RSI) and underwent videostroboscopy. Vocal function was objectively evaluated using perceptual ratings (GRBAS) and acoustic voice analysis (jitter, shimmer, maximum phonation time, maximum fundamental frequency range). The relationship between voice and swallowing symptoms was compared with thyroid volume and surgical pathology.

**Results and discussion:**

The average VHI score was 8.39, representing normal voice scores. Of the objective voice measures, only jitter and a subset of GRBAS measures were slightly elevated. Swallowing complaints were reported at least “some of the time” by 25 patients (41.7%), and “often or always” by 16 patients (26.7%). Of the patients with voice and swallowing complaints, 68.4% and 56%, respectively, had elevated RSI scores consistent with laryngopharyngeal reflux. No correlation was found with thyroid volume or thyroid pathology.

**Conclusions:**

Swallowing complaints appear to be common in pre-operative thyroidectomy patients. A large percentage, however, have associated laryngopharyngeal reflux which may in part account for these symptoms. Patients presenting with compressive thyroid symptoms should be evaluated for laryngopharyngeal reflux, and treated where appropriate.

## Introduction

Compressive symptoms, such as voice, swallowing and airway complaints thought to be secondary to a thyroid pathology, are an accepted indication for thyroidectomy (either subtotal or total thyroidectomy). Treating physicians frequently order thyroid investigations, including thyroid ultrasound, as a part of the work-up when patients present with these non-specific symptoms (globus sensation, throat discomfort, swallowing discomfort, voice changes etc.), often revealing the presence of previously undiscovered thyroid nodules. Whether these symptoms are indeed secondary to thyroid pathology or are due to a different process is often unclear. A review of the literature found the reported prevalence rates of voice and swallowing symptoms in pre-operative thyroidectomy patients to vary widely between 11-88% [1]-[7].

Although many of these studies report an improvement or resolution of compressive symptoms following thyroidectomy, a number of patients continue to experience voice and swallowing complaints post-operatively [4]-[8]. Due to the non-specific nature of these complaints, and the possibility of other underlying etiologies, the true prevalence of voice and swallowing symptoms due to thyroid pathology remains unclear. These same symptoms are common in the general population, and are often attributed to and relieved with treatment for laryngopharyngeal reflux (LPR) [9]-[11]. Currently there is very limited literature examining the prevalence of laryngopharyngeal reflux in thyroidectomy patients. To our knowledge, the only such study was performed by Fiorentino et al. [4], which found that 88% of patients undergoing thyroidectomy with compressive symptoms had pre-operative evidence of LPR on videofluoroscopic swallow studies (VFSS) [4]. All patients with evidence of LPR on VFSS pre-operatively continued to show changes consistent with LPR on VFSS post-operatively as well. More studies are certainly needed to further examine the prevalence of LPR among pre-operative thyroidectomy patients, and to determine its relationship to compressive symptoms.

The purpose of the current study is to determine the overall prevalence and severity of voice and swallowing complaints among preoperative thyroidectomy patients using both subjective and objective measures. In addition, we aim to determine the prevalence of laryngopharyngeal reflux in our cohort of preoperative thyroidectomy patients and to examine its relationship with compressive symptoms. A secondary objective was to determine if any correlation exists between thyroid volume or inflammatory thyroid pathology and the presence and severity of these symptoms.

## Methods

With approval of the Ethics Review Board at our institution, all patients diagnosed with a benign thyroid pathology or well-differentiated thyroid carcinoma (WDTC) who were consented to undergo a thyroidectomy procedure (subtotal or total thyroidectomy) over a 2 year period were invited to participate (October 2008-August 2011). When performed, a partial thyroidectomy consisted of a hemithyroidectomy plus isthmusectomy in all cases. Patients with a history of previous neck surgery (including previous thyroidectomy), prior head and neck radiation, medullary or anaplastic thyroid carcinoma, pre-operative recurrent laryngeal nerve dysfunction or other vocal fold pathology (evaluated using videostroboscopy) were excluded. In order to reduce the risk of age-related voice and swallowing complaints, no patients over the age of 75 years were included in this study. Data on patient factors, such as age, sex, indications for surgery, results of both fine-needle aspiration biopsy and final surgical pathology (including the presence of inflammation or thyroiditis within the specimen) and pre-operative ultrasound characteristics (total thyroid size; number and size of all thyroid nodules) were recorded. Total thyroid gland volume for each patient was calculated from pre-operative ultrasound measurements using the corrective factor 0.479, as recommended by the World Health Organization [12].

Subjective voice and swallowing complaints were assessed in each patient using the Voice Handicap Index (VHI) [13] and the modified Swallowing Impairment Score (SIS) [5], respectively. Objective acoustic voice measures ((jitter, shimmer, maximum phonation time (MPT), maximum fundamental frequency range (ST range) were collected in a standardized fashion and analyzed using the Multi-Dimensional Voice Program (MDVP) (Kay Elemetrics Version 1.34, 1993). All digital voice recordings were gathered in a sound-treated audiometric booth with each patient seated comfortably in an adjustable chair to maintain constant posture. Acoustic data was recorded with a head mounted microphone (AKG Acoustics, model C410) placed at a 90 degree angle to the left labial angle with the microphone end placed at 45 degrees to the corner of the mouth and out of the air stream (2 cm from the lip). Three voice tokens of the sustained vowel /a/ (at habitual pitch and loudness) were recorded for 5 seconds each, and the first 100 milliseconds of voice onset were trimmed from each sample to reduce variability. Each trimmed and isolated vowel token was analyzed for jitter (%) and shimmer (%). Maximum phonation time was collected by having the patient sustain the vowel /a/ at habitual pitch and loudness for as long as possible on a single breath following a maximal inhalation. Three trials were obtained, and the best result was retained for further analysis. Maximum fundamental frequency range was calculated in semi-tones and Hertz using the Voice Range Profile program from the Computerized Speech Lab. To calculate the maximum fundamental frequency (F0), the patient was asked to glide from a comfortably low note to their highest note on the vowel /u/. The best of three trials was recorded. Minimum fundamental frequency was calculated in the same manner was starting at a comfortably high note and gliding to the lowest note on the vowel /u/. The best of three trials was recorded. Glottal fry was omitted.

Perceptual voice analyses were performed on each patient by a certified Speech Language Pathologist trained in the diagnosis and treatment of voice disorders using the Grade, Roughness, Breathiness, Asthenia, Strain (GRBAS) rating scale [14]. All patients were required to complete the Reflux Symptom Index (RSI) [15] in order to evaluate the presence of concomitant laryngopharyngeal reflux. All data collection was performed in the pre-operative setting, either at the time of enrollment or at the same time as the patients’ pre-anaesthetic appointment in order to facilitate ease of data collection and limit additional hospital visits for our patients.

All statistical analyses were performed using SPSS Statistics Version 20 software. Pearson bivariate correlations using a two-tailed test were performed in order to identify correlations between variables. Jitter, shimmer, MPT and ST Range were compared with published norms using t-tests. Finally, a multivariate analysis of variance (ANOVA) was performed to identify independent relationships among variables. Statistical significance was defined as a p value less than 0.05.

## Results

Sixty one patients were invited to participate in this study. Two patients declined enrollment, leaving 59 patients (10 male, 49 female) in total ranging in age from 19–73 years (mean 48.8 years). Twenty four patients (40.7%) underwent a partial thyroidectomy thyroidectomy, and thirty five (59.3%) patients had a total thyroidectomy performed. The most common indication for surgery was a finding of atypical cells on fine needle aspiration biopsy (24/59), followed by nodule growth on successive ultrasounds (8/59). Six patients underwent a thyroidectomy procedure for the sole indication of compressive symptoms (Table 1). Thirty three patients (55.9%) were diagnosed with benign thyroid pathology, and 26 patients (44.1%) with well-differentiated thyroid carcinoma. Of the patients with a diagnosis of WDTC, 9 were reported as microscopic papillary thyroid carcinoma. The average total thyroid volume on ultrasound was 38.7 cm^3^ (range 6.3 – 237.9 cm^3^). There were no retrosternal thyroid goitres in this patient cohort. Patient characteristics, indications for surgery and final thyroid pathology are summarized in Table 1.

**Table 1:** **Patient demographics**

**Sex:**	
**Male**	**10**
**Female**	**49**
**Age:**	**48.6 years (19–73)**
**Surgery:**	
**Partial Thyroidectomy**	**24**
**Total thyroidectomy**	**35**
**Pathology:**	
**Benign**	**33**
-Multinodular Goitre	16
-Hyperplastic nodule	8
-Follicular/hurthle cell adenoma	7
-Graves disease	2
**Malignant (WDTC)**	**26**
**Indications for surgery:**	
Compressive symptoms	**6**
Multinodular goiter	**7**
Atypical cells	**21**
Follicular/Hurthle cell lesion	**4**
Nodule growth	**8**
Graves disease	**3**
Suspected WDTC	**4**
Known WDTC	**7**

Nineteen patients (32.2%) reported scores consistent with moderate to severe voice complaints on the VHI (9/59 moderate, 10/59 severe). All of the voice complaints were within the functional and physical domains, with no patients reporting elevated scores in the emotional domain. The average VHI score was 10.8, however, falling within the normal range. Objective acoustic voice analysis found jitter to be significantly elevated compared with normative data at 1.28 (p = 0.03); while shimmer was not significantly elevated. MPT and ST range were both within accepted norms (Table 2). Similarly, perceptual voice analysis using the GRBAS scoring system by a trained speech language pathologist did not reveal any significant abnormalities (G-0.52, R-0.62, B-0.33, A-0.16, S-0.35; p > 0.5).

**Table 2:** **Acoustic voice outcomes**

Variable	Pre-op thyroidectomy (mean)	Normative value (mean)	P-value
**Jitter (%)**	1.28	≤1.04	0.03*
**Shimmer (%)**	3.83	≤3.81	>0.05
**MPT (seconds)**	18.80	≥15	>0.05
**ST Range (dB)**	30.29	≥27	>0.05

Twenty five patients (42.7%) reported swallowing symptoms at least some of the time on the MSIS, with 16 patients (27.1%) reporting symptoms often or always. The average score on the MSIS was 6.5 out of a possible total score of 28, with the majority of symptoms relating to a globus sensation (Q5-7: “I always feel a sensation of lump in my throat”, “I feel a sensation of a lump in my throat when I lay down”, “I feel a sensation of a lump in my throat when I move my head”).

Twenty patients (33.9%) scored positive on the RSI for laryngopharyngeal reflux. Of the 19 patients with an elevated VHI, 68.4% (13/19) had an elevated RSI score consistent with LPR. Similarly, 56% (14/25) of patients with swallowing complaints also had scores on the RSI suggestive of laryngopharyngeal reflux. In addition, a positive correlation was found between MSIS and RSI scores (p < 0.001) indicating that those who scored highest with regards to swallowing complaints also scored highest for LPR.

When compared with thyroid volume on ultrasound, there was no significant correlation between VHI, jitter, shimmer, MPT, MSIS or RSI. There did appear to be a negative correlation between ST range and thyroid volume. That is, as thyroid volume increases, the number of semitones a person has in their range decreases (p = 0.045). No significant relationships were found between the presence of inflammatory thyroid pathology and any of the above measures (Table 3).

**Table 3:** **Effect of thyroid volume and inflammatory pathology**

	U/S thyroid volume (p value)	Inflammatory pathology (p value)
**VHI**	>0.05	>0.05
**Jitter**	>0.05	>0.05
**Shimmer**	>0.05	>0.05
**MPT**	>0.05	>0.05
**Semitone Range**	>0.045*	>0.05
**MSIS**	>0.05	>0.05
**RSI**	>0.05	>0.05

*Negative correlation.

## Discussion

The prevalence of self-reported voice and swallowing complaints in our cohort was found to be 32.2% and 42.7%, respectively. A literature review found that the reported prevalence of compressive symptoms in pre-operative thyroidectomy patients varied widely between studies, ranging from 11-88% [1]-[7]. Unfortunately many of these studies vary with regards to the symptoms reported as well as the methods used for data collection (e.g. patient interviews, visual analogue scales, subjective patient questionnaires), making comparisons between cohorts difficult.

Despite 32.2% of patients reporting moderate to severe voice complaints on the VHI, the overall mean score of 10.8 was not significantly elevated (although trending toward moderate dysfunction). Objective acoustic voice analysis showed only jitter to be significantly elevated in our cohort at 1.28%. In addition, perceptual analysis by a trained Speech Language Pathologist using the GRBAS scoring system did not reveal significant abnormalities in voice quality, despite patient perceptions of dysfunction. This is in agreement with the results from several studies where jitter, shimmer, MPT, ST range, and GRBAS measures were all within the normal range; often despite patient complaints of voice abnormalities [5],[6],[16],[17]. In addition, studies have shown that 14-75% of patients continue to complain of voice symptoms 3 months −1 year post thyroidectomy, questioning the origin of these symptoms with regards to thyroid disease [4]-[6],[16].

Swallowing dysfunction is a commonly reported symptom in pre-operative thyroidectomy patients, with the majority complaining of a globus sensation [1],[3]-[5],[7],[18]. In our cohort, 47.2% of patients reported swallowing symptoms at least some of the time, with 27.1% reporting symptoms often or always; with the majority of symptoms relating to a globus sensation. Although many studies do show improvement in swallowing complaints post-thyroidectomy, a variable proportion of patients continue to report symptoms 3 months to 1 year post-thyroidectomy (20-91%) [3]-[5],[18].

Given the fact that there does appear to be a subgroup of pre-operative thyroidectomy patients with voice and swallowing complaints that does not respond to thyroidectomy, we questioned the possible co-existence of laryngopharyngeal reflux in these patients. As such, we administered the RSI to our patients and found that 33.9% of pre-operative thyroidectomy patients had concomitant laryngopharyngeal reflux, which is in keeping with reported LPR rates in the general population [9]-[11]. However, when we looked specifically at the relative contribution of LPR to voice and swallowing complaints in this cohort we found that laryngopharyngeal reflux may account for a larger proportion of patients with elevated symptom scores. That is, 68.4% of patients with elevated VHI scores, as well as 56% of patients with swallowing complaints on the MSIS also scored positive for LPR. To our knowledge, the only other study to date to look at LPR in thyroidectomy patients is Fiorentino et al. [4], who found that 88% of pre-operative thyroidectomy patients with compressive symptoms also had pre-operative evidence of LPR on videofluoroscopic swallow studies (VFSS) [4]. In addition, all patients with evidence of LPR on VFSS pre-operatively continued to show changes consistent with LPR on VFSS post-operatively as well. Further studies are needed in order to explore the relationship between compressive symptoms and LPR, as well as to determine the impact of reflux treatment on post-thyroidectomy symptomatology.

## Conclusions

Compressive symptoms, such as voice and swallowing complaints, are an accepted indication for thyroidectomy (either total or partial). This study shows the prevalence of voice and swallowing complaints in pre-operative thyroidectomy patients to be 32.2% and 42.7%, respectively. In addition, we show that a large percentage of patients presenting with voice and swallowing complaints may have associated laryngopharyngeal reflux, which can at least in part account for these symptoms. As such, all patients presenting with compressive thyroid symptoms should also be evaluated for laryngopharyngeal reflux, and treated where appropriate prior to performing a thyroidectomy.
